# The rehabilitation efficacy of diaphragmatic breathing combined with limb coordination training for lower limb lymphedema following gynecologic cancer surgery

**DOI:** 10.3389/fbioe.2024.1392824

**Published:** 2024-06-06

**Authors:** Jingxin Wang, Jiahui Ma, Yujie Zhang, Yuan Tian, Xinxin Wang, Yu Wang, Dongquan Xiang, Daoyu Wang, Kun Huang, Luxi Mao, Jiaxin Zhang, Huixuan Fan, Yilan Li

**Affiliations:** ^1^ Department of Rehabilitation Medicine, Zhengzhou Central Hospital Affiliated Zhengzhou University, Zhengzhou, China; ^2^ Xinxiang Medical University, Xinxiang, China; ^3^ Department of Geriatrics, The First Affiliated Hospital of Fujian Medical University, Fujian Key Laboratory of Molecular Neurology and Institute of Neuroscience, Fujian Medical University, Fuzhou, China; ^4^ Department of Ultrasound Medicine, Zhengzhou Central Hospital Affiliated Zhengzhou University, Zhengzhou, China; ^5^ Fuwai Central China Cardiovascular Hospital, Zhengzhou, China; ^6^ Senior Department of Orthopedics, The Fourth Medical Centre, Chinese PLA General Hospital, Beijing, China; ^7^ Academy for Engineering and Technology, Fudan University, Shanghai, China

**Keywords:** diaphragmatic breathing, coordination training, rehabilitation efficacy, lower limb lymphedema, gynecologic cancer surgery

## Abstract

**Objective:**

To investigate the impact of diaphragmatic breathing combined with limb training on lower limb lymphedema following surgery for gynecological cancer.

**Methods:**

From January 2022 to May 2022, 60 patients with lower limb lymphedema post-gynecologic cancer surgery were chosen. They were split into a control group (*n* = 30) and a treatment group (*n* = 30). The control group underwent complex decongestive therapy (CDT) for managing lower limb lymphedema after gynecologic cancer surgery, while the treatment group received diaphragmatic breathing combined with limb coordination training alongside CDT. Both groups completed a 4-week treatment regimen. The lower limb lymphedema symptoms were evaluated using the genital, lower limb, buttock, and abdomen (GCLQ) scores; bilateral lower limb circumference measurements; and anxiety and depression scores.

**Results:**

Compared to sole CDT administration, individuals undergoing diaphragmatic breathing coupled with limb coordination training experienced notable reductions in scores for the self-perceived symptom assessment questionnaire (GCLQ), bilateral lower limb circumference, as well as anxiety and depression scores.

**Conclusion:**

The incorporation of diaphragmatic breathing combined withalongside limb coordination training can accelerate and augment the efficacy of treating lower limb lymphedema post-gynecologic cancer surgery.

## 1 Introduction

In recent years, the prevalence of lower limb lymphedema following gynecologic cancer surgery has steadily risen, mirroring the high occurrence of gynecologic malignancies, which ranges from 20% to 60% (Carlson et al., 2020). Common gynecologic cancers include cervical cancer, ovarian cancer, endometrial cancer, and vaginal cancer, among others. Surgical procedures often entail pelvic lymph node dissection, while varying degrees of chemotherapy and radiotherapy postoperatively can impair the lymphatic system responsible for draining lymphatic fluid from the lower limbs (Kalemikerakis et al., 2021; Zeng et al., 2021; Dokku et al., 2023; Patil et al., 2023). Moreover, postoperative factors such as obesity, insufficient physical activity, and unhealthy habits can impede lower limb lymphatic circulation, resulting in swelling, discomfort, and sensory disturbances in the lower extremities (Yildiz Kabak et al., 2021; Sudduth and Greene, 2022; Faerber, 2023). Severe cases may lead to complications such as cellulitis, acute infections, and can trigger anxiety, and depression, significantly impacying patients’ quality of life and occupational functioning (Dessources et al., 2020; Tedeschi, 2023).

Previously, complex decongestive therapy (CDT) was extensively employed as a cornerstone treatment for lymphedema (Executive Committee of the International Society of Lymphology, 2020; Liu et al., 2021). CDT involves manual lymphatic drainage, skin care, compression bandaging, and physical exercise. However, its effectiveness is limited and requires sustained patient adherence. With the increasing diversity of patients and the expanding range of clinical interventions, numerous novel approaches are now being integrated into clinical practice (Thomas et al., 2020). Studies indicate that following standardized deep breathing (Douglass et al., 2020), there is a notable immediate reduction in limb volume and edema percentage, persisting for at least 30 min. Diaphragmatic breathing enhances respiratory muscle strength, endurance, and coordination, improves chest mobility, fortifies thoracic negative pressure capacity, and fosters deep blood and lymph circulation; thus, enhancing lymphatic flow (Fröhlich, 2021). Physical exercise regulates rhythmic compression of blood vessels and lymphatic systems, sustaining optimal muscle contraction strength to emulate a “muscle pump” effect, thereby aiding in augmenting lower limb lymphatic return and alleviating lymphedema (Hasenoehrl et al., 2020; Tendero-Ruiz et al., 2020; Saraswathi et al., 2021; Aguilera-Eguía et al., 2023).

The limb coordination training device LoopGO supports the gradual restoration of lower limb control, coordination, strength, joint range of motion, and other motor functions. In particular, it enhances patients’ autonomy, and compliance, and promotes standardized and safe functional exercise. This study focused on gynecologic cancer surgery patients with lower limb dysfunction, and compared the effects of the LoopGO with those of conventional rehabilitation techniques. The aim of this study was to provide new rehabilitation measures for patients with gynecologic cancer surgery and lower limb dysfunction.

## 2 Materials and methods

### 2.1 General data

Sixty instances of lower limb lymphedema following after gynecologic cancer surgery treated at our facility between January and May 2022 were included in the study. Inclusion criteria involved: 1) a history of lymph node dissection due to gynecologic malignancy, confirmed both clinically and through imaging for lower limb lymphedema; 2) unilateral lower limb edema; 3) absence of deep vein thrombosis verified by color Doppler ultrasound; 4) capability to complete a 4-week treatment regimen; 5) voluntary participation with signed informed consent signed. Exclusion criteria consisted of: 1) patients with acute skin infections like cellulitis and erysipelas, deep vein thrombosis, or severe cardiac conditions; 2) patients with suspected or confirmed cancer recurrence or metastasis; 3) patients unable to adhere to the treatment requirements during the study period.

We initially collected 122 patients, but after applying inclusion and exclusion criteria and considering other factors such as attrition, only 60 patients were ultimately eligible for treatment and analysis. This study utilized a single-blind experimental design, with researchers aware of the group allocations while subjects remained unaware of whether they belonged to the experimental or control group. Patient numbering and random selection were employed to assign 30 individuals to the treatment group and the remaining to the control group. Prior to study commencement, all trial implementers underwent centralized training to familiarize themselves with the study’s significance and procedures, participating only after passing the training assessment. Treatment was administered by certified rehabilitation therapists with extensive experience. No significant differences were observed between the two groups concerning age, disease type, postoperative edema duration, and other factor (*p* > 0.05), indicating their comparability. Please refer to Table 1. All procedures conducted in this study received approval from the Medical Ethics Committee of Zhengzhou Central Hospital (No. 202256).

**TABLE 1:** General Information of the Two Patient Groups. ^(1)^ *t*-test; ^2)^ Fisher’s exact test.

	Control Group (*n* = 30)	Treatment Group (*n* = 30)	*p*-Value
Age (Years)	55.03 ± 9.74	54.1 ± 8.27	0.691^1)^
Cancer type			0.446^2)^
Cervical Cancer (Number of Cases)	20 (66.7%)	18 (60%)	
Endometrial Cancer (Number of Cases)	7 (23.3%)	10 (33.3%)	
Ovarian Cancer (Number of Cases)	3 (10%)	1 (3.3%)	
Fallopian Tube Cancer (Number of Cases)	0	1 (3.3%)	
Postoperative Edema Duration (Years)	5.33 ± 1.75	5.13 ± 1.87	0.604^1)^

### 2.2 Experiments design

#### 2.2.1 Control group

Each patient in this group underwent CDT, which included the following (Schiltz et al., 2024): ① Manual Lymphatic Drainage (MLD): According to the Vodder method created by Dr. Vodder in the third edition of Foldi’s Lymphology, lymph node activation and lymphatic drainage were performed at the required treatment sites, with 5-7 activations and drainages at each site. MLD was conducted once daily for 40 min each time. ② Pressure Therapy: This involves Intermittent Pneumatic Compression (IPC) and Bandaging Pressure Therapy. Using the Israeli Megoafek company’s 24-cavity type III air wave pressure therapy machine, pressure therapy was applied to the patients’ bilateral lower limbs and waist-abdomen areas before and after MLD. IPC mimics manual lymphatic drainage, reaching areas inaccessible to manual techniques, with a treatment intensity set at 55 mmHg, administered once daily for 40-min sessions. Following MLD and IPC treatment, bandaging pressure therapy is applied. Various widths of German Biaform low-stretch bandages (6-9 rolls) are applied in an “eight” pattern to exert pressure on the affected leg and foot once daily for 24 h, including overnight during sleep. ③ Skin care involves the uniform application of Vitamin E cream to the affected lower limb before and after bandaging removal to maintain soft and smooth skin. Daily skin cleansing and hygiene are vital, including cleaning or wiping the affected lower limb before each skin care session. ④ Edema exercise includes passive supine bilateral lower limb isometric training conducted once daily for 20 min following bandaging pressure. Each patient undergoes these treatments continuously for 4 weeks.

#### 2.2.2 Treatment group

In the treatment group, patients underwent diaphragmatic breathing along with limb coordination training, in contrast to the control group. Before starting CDT treatment, breathing training occurred with patients lying supine and knees bent. Therapists placed their hands on the patient’s abdomen, offering resistance based on their ability to maintain diaphragmatic breathing. Meanwhile, patients were instructed to practice resistance diaphragmatic breathing while pursing their lips, inhaling through the nose, and exhaling slowly as if blowing out a candle. The inhalation-exhalation ratio remained at 1:2, with each group completing five breaths, totaling two sets before initiating CDT treatment. Each training session lasted for 10 min. Conversely, the control group continued normal breathing throughout this period.

**FIGURE 1:**
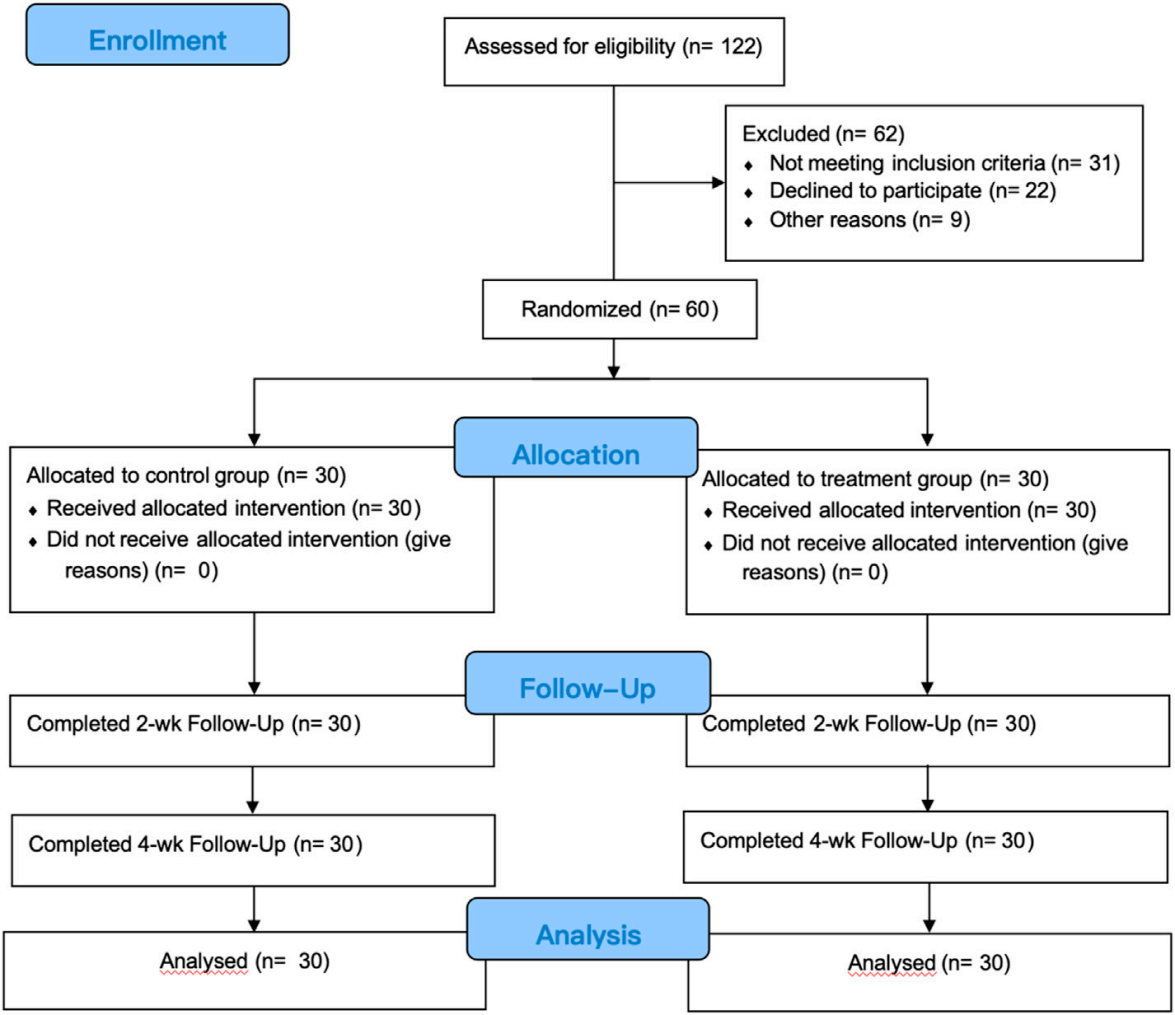
Flow chart of study implementation. Control group: received complex decongestive therapy; Treatment group: received complex Decongestive therapy, Diaphragmatic Breathing and Coordination Training.

**FIGURE 2:**
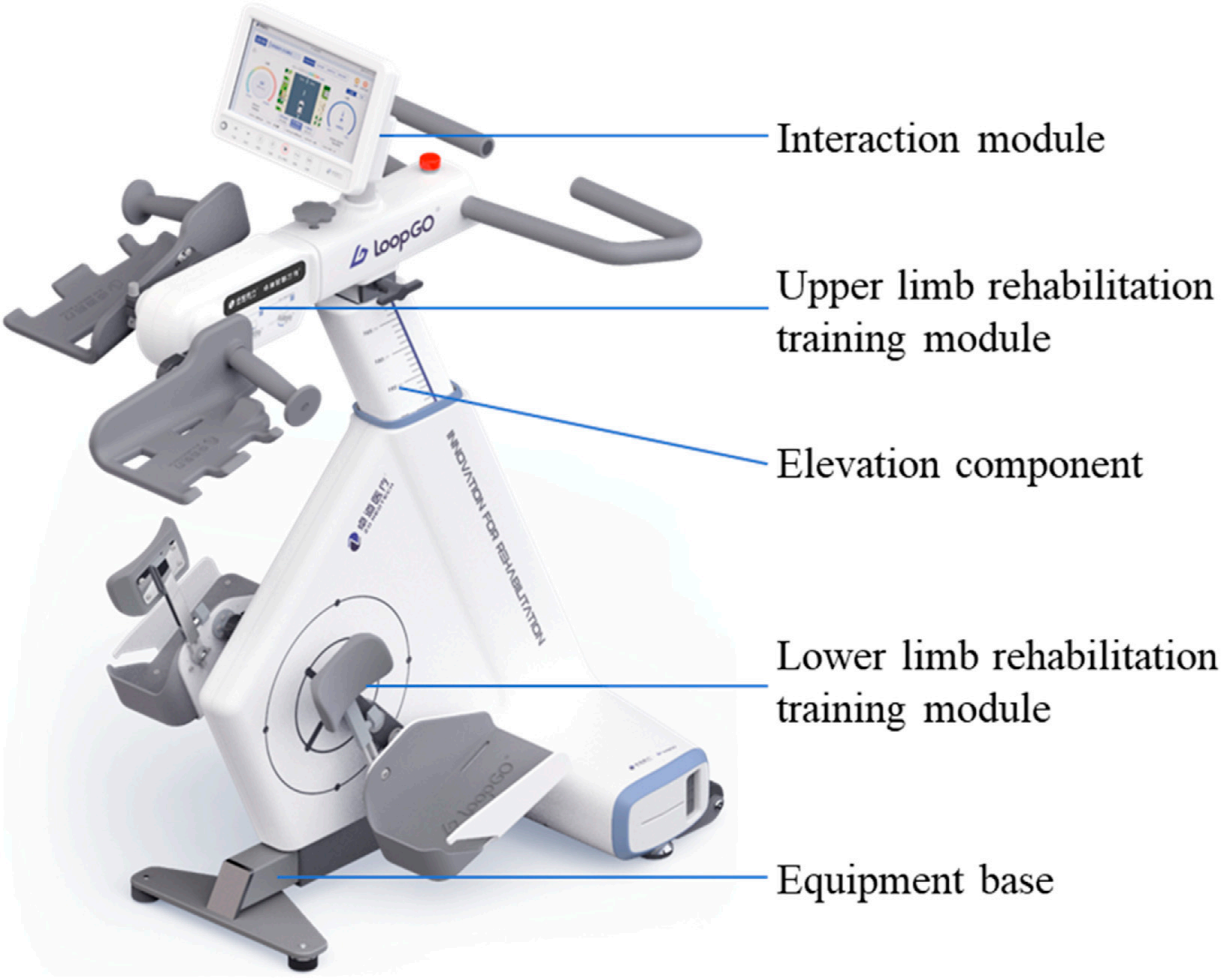
The prototype of the limb coordination training device.

Limb coordination training was initiated on the lower limb of the affected side after CDT treatment, using a limb coordination training device LoopGO (see Figure 2). The prototype of the limb coordination training device is shown in Figure 1. The LoopGO includes upper and lower limb training modules. We selected a multihead worm gear reducer as the power source for the upper limb training module. To achieve synchronous or asynchronous circular motion with both hands securely fixed, the above mentioned worm gear reducer mentioned above should be a dual-output shaft reducer. The upper limb training module requires two crank components, a driving crank and a gripping crank. The gripping crank must rotate freely relative to the crank. Both crank components should be adaptable to mount in reverse or forward orientations on the output shafts positioned on both sides of the worm gear reducer. The lower limb training module has opted for a motor with an output end of a Φ16 mm multi-vee pulley as the power source. The motor, driven by a multi-vee belt, rotates a large-diameter turntable through speed reduction and torque amplification, ensuring that the maximum motion torque of the turntable is no less than 20 Nm.

The standard training program involved activating the lower limbs primarily. Patients were guided to sit and exert force with both legs together while maintaining a neutral body position. Each training session lasted for 20 min and was conducted once daily, consistently over a span of 4 weeks.

### 2.3 Evaluation standard

#### 2.3.1 Lower limb lymphedema self-perceived symptom assessment questionnaire (GCLQ)

To assess the GCLQ scores (Carter et al., 2019) of the two groups before and after the 4-week intervention, we employed the Lower Limb Lymphedema Self-Perceived Symptom Assessment Questionnaire (GCLQ). This questionnaire was used to evaluate sensations associated with lower limb lymphedema experienced over the past 4 weeks. It comprises seven symptom clusters, including heaviness, overall edema, local swelling, sensory symptoms, pain, numbness, and limb function, totaling 20 items. Each item is rated on a scale of 0 to 1, where 0 indicates the absence and 1 indicates the presence of the symptom. A total score of ≥4 signifies the diagnosis of lower limb lymphedema. The questionnaire exhibits good sensitivity (92.86%) and specificity (83.33%) when the total score is ≥4.

#### 2.3.2 Bilateral lower limb circumference measurement

The bilateral lower limb circumferences of the two groups of patients before and after the 4-week intervention were compared, and the circumferences of the ankles at the narrowest point cB, the widest point of the calf cC, and the root of the thigh cG on the affected side were measured with a tape measure.

#### 2.3.3 Hospital anxiety and depression scale

Comparison of the Hospital Anxiety and Depression Scale (HADS) (Annunziata et al., 2020) scores before and after the 4-week intervention demonstrated significant changes. Originally introduced by Zigmond and Snaith in 1983, the HADS is predominantly used by healthcare professionals to assess anxiety and depression severity in hospitalized patients. It comprises 14 items, equally divided between depression and anxiety. Each item rates the frequency of symptoms over the past month on a 4-point scale, ranging from 0 to 3 points resulting in four severity levels. The maximum score for both anxiety and depression is 21 points, with higher scores indicating more severe symptoms. Scores are categorized as follows: 0–7 points (negative), 8–10 points (mild), 11–14 points (moderate), and 15–21 points (severe).

### 2.4 Statistical analysis

#### 2.4.1 Sample size

We used G*Power (version 3.1) to estimate the sample size required to detect differences in the effects of group × time interactions on clinical outcomes (CCLQ score, circumference of the lower limbs, and HADS score). We mainly used F tests for specific analyses, and ANOVA: Repeated measures, and within-between interactions were used in the statistical test. By default, the effect size f is 0.25, the 2-sided significance level is 0.05 and the power is 95%. Based on this analysis, the target sample size was 54.

We organized the data and conducted statistical analyses using SPSS and Prism9. The final results are presented as the mean ± standard error of the mean (SEM). ANOVA was employed to assess the effect of different conditions on the treatment. Differences between pre-treatment, 2-week, and 4-week intervals were evaluated through two-sided paired t-tests. SPSS was utilized for analysis by Pearson Chi-square, Likelihood Ratio Chi-square or Fisher’s exact test. Statistical significance was determined with a *p*-value of <0.05.

## 3 Results

### 3.1 Lower limb lymphedema self-perceived symptom assessment questionnaire (GCLQ)

**FIGURE 3:**
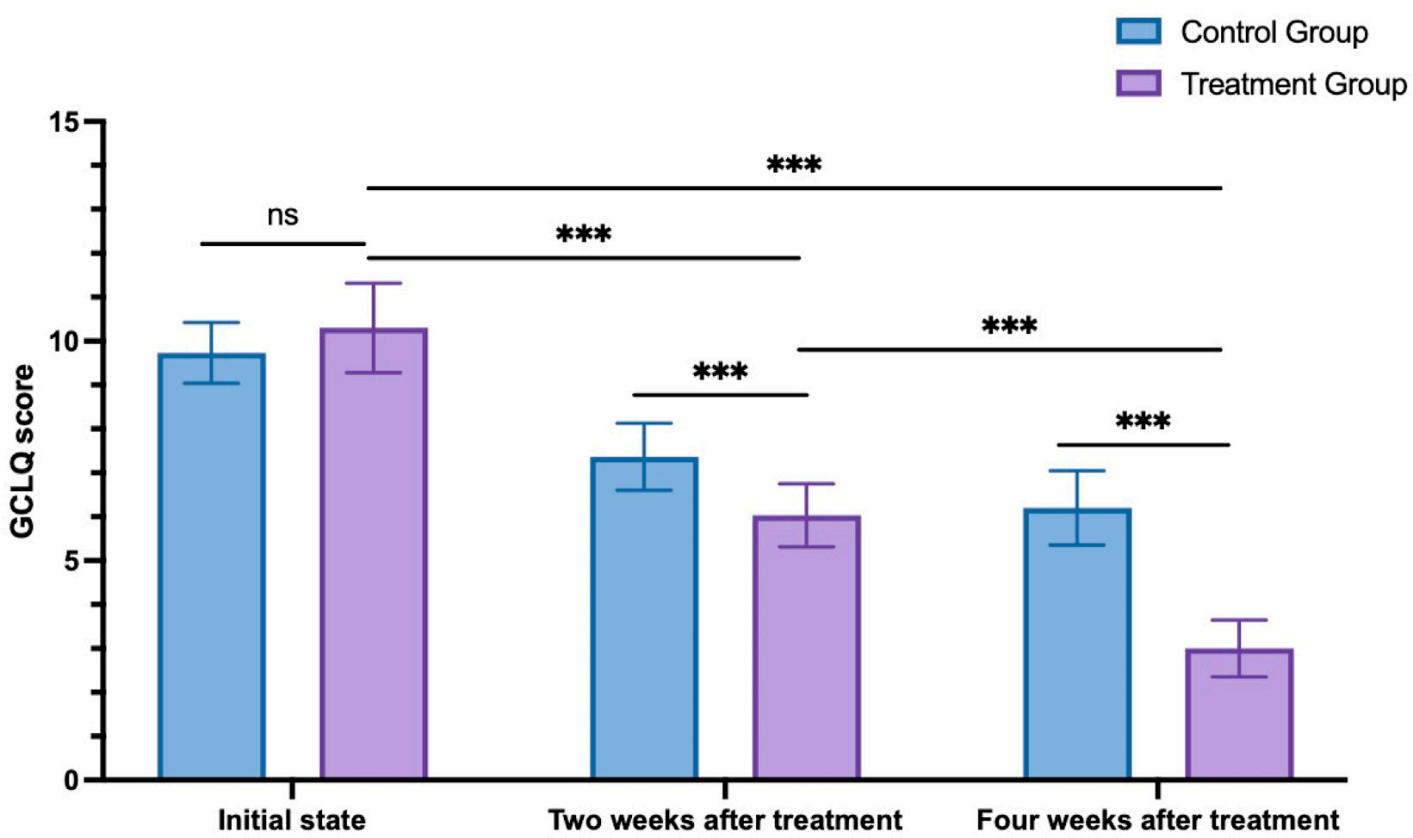
GCLQ score changes in the control group and treatment group. ns: non-significant; ***p < 0.001.

For the control group, the GCLQ scores were 9.73 ± 0.69 at the initial state, 7.37 ± 0.77 2 weeks after treatment, and 6.20 ± 0.85 4 weeks after treatment. Meanwhile, for the treatment group, the GCLQ scores were 10.30 ± 1.02 at the initial state, 6.03 ± 0.72 2 weeks after treatment, and 3.00 ± 0.64 4 weeks after treatment. Two-way ANOVA was used to compare the changes in GCLQ scores between the two groups at different time points. The results indicated no significant difference between the two groups at the initial state (*p* = 0.067). As time progressed, both the 2-week and 4-week posttreatment scores were significantly lower than the initial scores for both groups. Furthermore, at both the 2 weeks after treatment and 4 weeks after treatment, the treatment group exhibited significantly lower scores than the control group (*p* < 0.001, see Figure 3).

### 3.2 Bilateral lower limb circumference measurement

**FIGURE 4:**
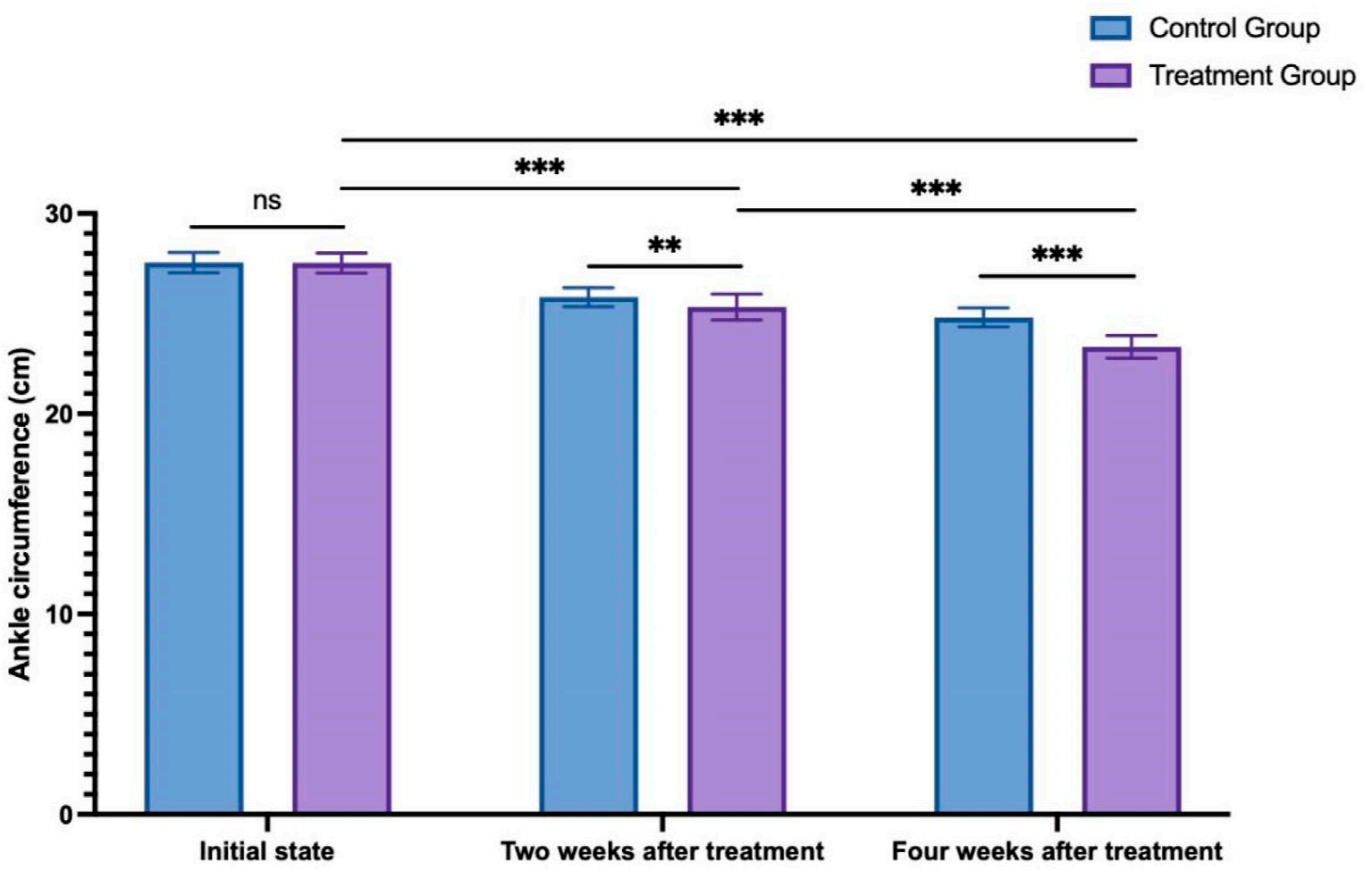
The change in ankle circumference between the control group and treatment group. ns: non-significant; **p < 0.01; ***p < 0.001.

The initial ankle circumference, measured at 2 weeks and 4 weeks after treatment in the control group, was 27.55 ± 0.51 cm, 25.82 ± 0.48 cm, and 24.81 ± 0.48 cm, respectively. In the treatment group, the initial ankle circumference, 2 weeks, and 4 weeks after treatment were 27.53 ± 0.50 cm, 25.33 ± 0.64 cm, and 23.34 ± 0.57 cm, respectively. A two-way ANOVA analysis compared changes in bilateral lower limb circumference of both lower limbs between the two groups at different time points. Initially, there was no significant difference between the two groups at the initial state (*p* > 0.999). However, as time progressed, both groups showed significantly lower measurements at 2 and 4 weeks after treatment compared to the initial state (*p* < 0.001). Additionally, at both 2 weeks after treatment (*p* = 0.006) and 4 weeks after treatment (*p* < 0.001), the treatment group exhibited significantly lower measurements compared to the control group (see Figure 4).

### 3.3 Hospital anxiety and depression scale

**FIGURE 5:**
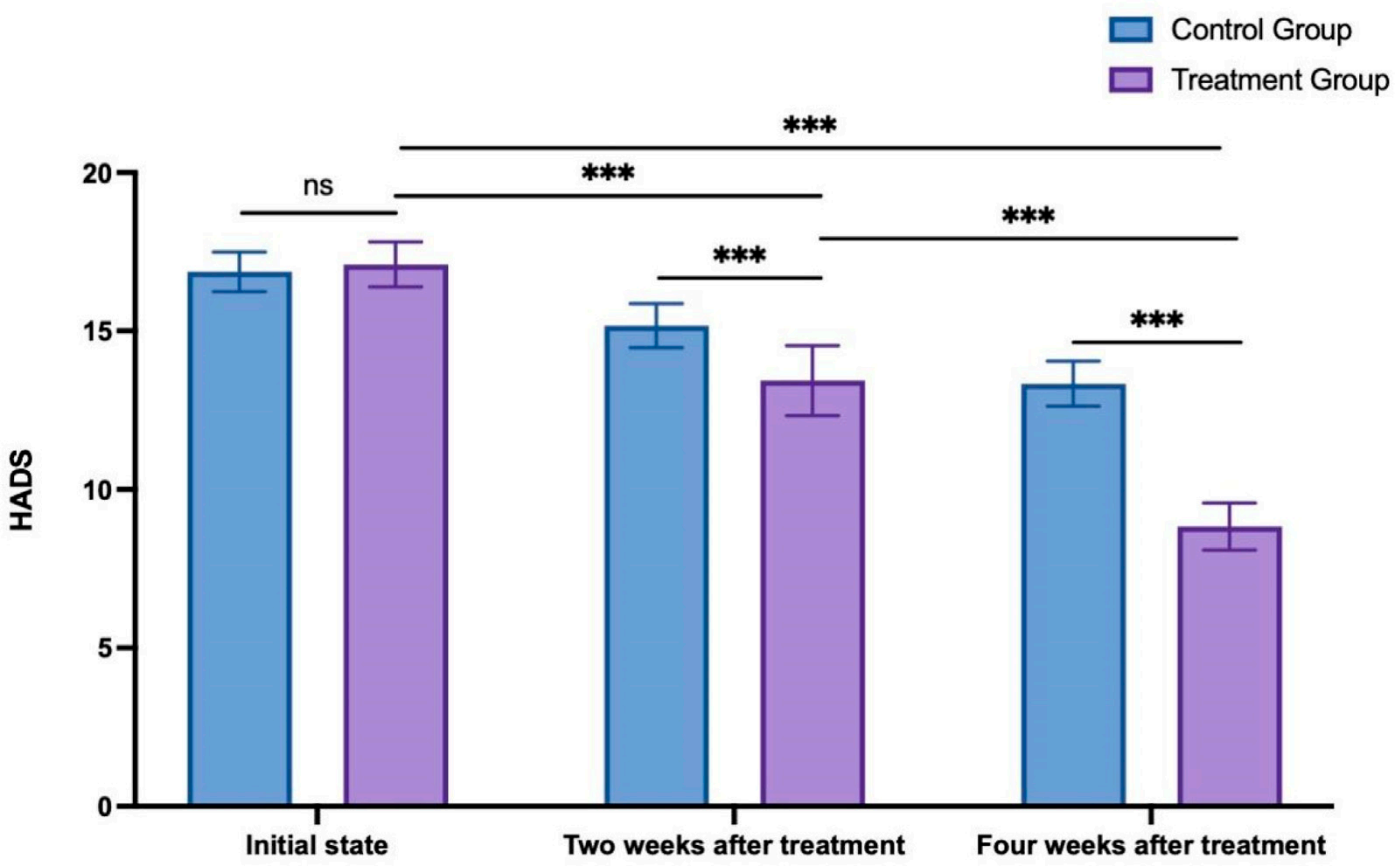
HADS score changes in the control group and treatment group. ns: non-significant; ***p < 0.001.

The HADS scores (Blumenthal et al., 2021) recorded at the initial state, 2 weeks after treatment, and 4 weeks after treatment in the control group were 16.87 ± 0.63, 15.17 ± 0.70, and 13.33 ± 0.71, respectively. Meanwhile, in the treatment group, the HADS scores at the initial state, 2 weeks after treatment, and 4 weeks after treatment were 17.10 ± 0.71, 13.43 ± 1.10, and 8.83 ± 0.75, respectively. Analysis using two way ANOVA analysis was conducted to compare the alterations in HADS scores between the two groups at various time points. The results indicated no significant difference between the two groups in the initial state (*p* = 0.857). As time progressed, both the 2-week and 4-week posttreatment scores were significantly lower than the initial scores for both groups. Furthermore, at both the 2 weeks after treatment and 4 weeks after treatment, the treatment group exhibited significantly lower scores than the control group (*p* < 0.001, see Figure 5).

## 4 Discussion

While studies on lower limb lymphedema post-gynecological cancer surgery are less abundant compared to those on secondary upper limb lymphedema, they demonstrate a consistent trend of progression and treatment challenges. Although early-stage lower limb lymphedema may temporarily improve after a night’s rest, it typically advances as a progressive condition often disregarded by patients in its initial phases, leading to missed opportunities for optimal treatment. This progression can culminate in irreversible stages like skin fibrosis, elephantiasis, and severe complications such as skin infections, significantly impacting patients’ daily lives, work, and mental well-being. Consequently, it imposes a considerable psychological burden and anxiety on affected individuals (Săvulescu et al., 2021; Baik et al., 2022; Lee et al., 2022).

During rest, lymphatic flow occurs at a slow pace, with interstitial fluid entering lymphatic capillaries to form lymph. Following a series of filtration and convergence processes, lymph proceeds to the thoracic duct or right lymphatic duct before eventually entering the bloodstream through the superior vena cava system. Under normal physiological circumstances, there exists a dynamic equilibrium between lymphatic load and transport capacity. However, gynecologic cancer surgeries or treatments such as radiation and chemotherapy can disrupt normal lymphatic tissue, resulting in inadequate transport capacity, accumulation of interstitial fluid in the subcutaneous tissue, and the onset of lymphedema (Bartels et al., 2023; Daggez et al., 2023). Various factors contribute to the driving force behind lymphatic flow including muscle contractions, intrathoracic negative pressure, lymphatic pump, and arterial pulsation. During inspiration, the increased intrathoracic negative pressure promotes dilation of the thoracic duct, thereby facilitating lymphatic flow. Studies have demonstrated that in dogs subjected to excessive ventilation, thoracic duct flow increases, whereas it decreases when the thoracic cavity is opened and intrathoracic pressure is reduced. Human trials conducted by Ajima revealed that 30 min of diaphragmatic breathing in a supine position effectively induced thoracic duct lymphatic drainage (Kawai et al., 2015). Furthermore, Ratnayake et al. (Ratnayake et al., 2018) observed that the pressure difference between the terminal lymphatic duct and the venous end varies with the respiratory cycle. Hence, respiratory movements can effectively facilitate gas exchange in the alveoli, diminish venous hypertension, and induce lymphatic fluid flow. In 2020, the International Society of Lymphology’s consensus statement underscored the significance of integrating deep breathing training into self-management strategies for breast cancer-related lymphedema (BCRL) (Wang et al., 2023). Additionally, the Clinical Practice Guidelines (CPG) developed and released by the Oncology Section of the American Physical Therapy Association advocate for a range of measures, including deep breathing exercises, to ameliorate lymphedema while promoting relaxation and enhancing emotional well-being among patients (Campione et al., 2023).

Active exercise plays a crucial role in the rehabilitation of lymphedema, while resistance training involves engaging in rhythmic, multi-set weight or resistance exercises to stimulate muscle contraction (Zaborova et al., 2023). This stimulation augments muscle pressure, thereby facilitating lymphatic drainage, enhancing metabolic capacity, and alleviating lymphedema symptoms. Limb coordination training adjusts the training mode and intensity according to the patient’s strength, thereby regulating the training intensity by modifying resistance levels. By exerting pressure on deep tissues, this training encourages muscle contractions and relaxation, effectively acting as a “muscle pump” to facilitate the return of lymphatic fluid.

The study results show that combining diaphragmatic breathing with limb coordination training can reduce lower limb lymphedema following gynecologic cancer surgery. This combination significantly enhances lower limb circumference, emotional well-being, and symptom perception compared to the control group. Thus, focusing on patients’ breathing conditions and integrating complex decongestive therapy after respiratory training can improve rehabilitation outcomes when combined with limb coordination training.

The study has limitations. The sample size might restrict the generalizability of the findings, necessitating larger studies to endorse the conclusions. Additionally, the investigation focused solely on the 4-week treatment duration, warranting further exploration of the long-term effects of diaphragmatic breathing and limb coordination training.

## 5 Conclusion

In conclusion, diaphragmatic breathing exhibits superior effectiveness in treating lower limb lymphedema after gynecologic cancer surgery compared to traditional CDT therapy. Future clinical practice, should prioritize diaphragmatic breathing and limb coordination training. This approach is safe, yields visible outcomes, reduces treatment duration for patients, and warrants dissemination among therapists specializing in post-gynecologic cancer surgery lower limb lymphedema treatment.

## Data Availability

The raw data supporting the conclusion of this article will be made available by the authors, without undue reservation.
